# Supplementary material for: EpiCHAOS: a metric to quantify epigenomic heterogeneity in single-cell data
Source: Genome Biol. 2024 Dec 3;25:305. doi: 10.1186/s13059-024-03446-w (PMC11613708; doi:10.1186/s13059-024-03446-w)
Supplement: Supplementary file 4 — Additional file 4: Review history. [file 13059_2024_3446_MOESM4_ESM.docx]

Review History

**First round of review**

**Reviewer 1**

**Were you able to assess all statistics in the manuscript, including the appropriateness of statistical tests used?**

No.

**Were you able to directly test the methods?**

Yes.

**Comments to author:**

Kelly et al. proposed a concise metric, epiCHAOS, to evaluate epigenomic heterogeneity in certain single-cell clusters or groups, and applied this metric to multiple single-cell datasets to associate it with cellular phenotypes, such as stemness and plasticity, and genomic features. Although epiCHAOS reflects certain associations with some biological features, there are some limitations which should be addressed when it is widely used in single-cell data routine analyses in future.
Major
1. Although epiCHAOS has been applied in many single-cell datasets, the conclusions are highly dependent on prior knowledge. Are there any examples that we could gain new insights into some biological processes by utilizing epiCHAOS? It is more meaningful that a method could solve some problems or bring some inspirations when used in single-cell routine analyses.
2. A epiCHAOS score is calculated for one single-cell cluster or group. It means that this metric is largely dependent on the clustering precision of single cells. However, the single cell clustering is unsupervised and determined by experience. When a large cell cluster is further clustered into some more homologous sub-clusters, will epiCHAOS alter? How to balance epiCHAOS and clustering precision?
3. In this manuscript, the authors tended to compare epiCHAOS scores between different cell clusters. In another dimension, they could compare epiCHAOS scores between different samples within the same single-cell clusters or cell types. For example, analyzing datasets associated with aging or drug sensitivity or disease severity.
Minor
4. P values should be provided for regression or correlation in Fig. 2D-F, Fig3. B-C, Fig4A, D, Fig. 5B.
5. Another possible utilization for epiCHAOS: could epiCHAOS score used for guiding single cell clustering and evaluating the similarity across different clusters?
6. It is difficult to determine the difference significance between two clusters by solely comparing two epiCHAOS scores. The authors could introduce P values when comparing two clusters in the method. A possible solution is single cell shuffling across clusters.

**Reviewer 2**

**Were you able to assess all statistics in the manuscript, including the appropriateness of statistical tests used?**

**Yes:** The use of statistics is appropriate.

**Were you able to directly test the methods?**

No.

**Comments to author:**

The paper provides a novel metric to measure epigenetic heterogeneity, the epiCHAOS score. This score is calculated as the mean pairwise distance (e.g. the Jaccard distance for binary scATAC-seq) between cells within a defined population (e.g., a cell cluster), representing the average dissimilarity or heterogeneity among the cells in that cluster. This score is then adjusted for technical factors, such as differences in genome-wide chromatin accessibility, to ensure that the heterogeneity reflects biological variation regardless of sequencing depth. The validation of epiCHAOS across various contexts—including less differentiated, multipotent cell states in human hematopoiesis, mouse gastrulation, and Drosophila embryogenesis; breast and liver cancer patients; and specific gene sets—demonstrates a comprehensive and well-rounded biological argument of linking epigenetic heterogeneity to key biological processes across different contexts. The ability of epiCHAOS to reveal elevated heterogeneity at PRC2 targets, CTCF, and Cohesin binding sites further strengthens its application in understanding epigenetic regulation. Some comments are still needed, and are listed below:

1. The current approach of generating heterogeneous datasets (Fig 1B) (by incrementally removing a set number of "1's" from selected rows and adding them to different rows) introduces a circular problem. Since the Jaccard distance compares the number of shared elements to the total number of unique elements across the sets, manipulating the matrix in this way could artificially influence the metric, potentially leading to results that reflect the method's design rather than real-world biological variations. It would be more effective to focus on in silico simulations that incorporate more biologically relevant models, or by adapting tools for simulation that better mimic the diversity and complexity of real-world data.
2. To simulate biological samples with varying sequencing depth (Fig. 1C), it could be valuable to adopt more sophisticated scATAC simulation strategies / tools, such as scReadSim. This will generate synthetic data that more closely mimics real sequencing conditions, incorporating realistic variations in read counts, sequencing errors, and chromatin accessibility distribution patterns. For instance, varying sequencing depth in simulations can better reflect how differences in coverage impact peak detection and data quality.
1. Yan, G., Song, D. & Li, J.J. scReadSim: a single-cell RNA-seq and ATAC-seq read simulator. *Nat Commun* **14**, 7482 (2023). https://doi.org/10.1038/s41467-023-43162-w
3. In Fig 4B, the PRC2 targets (EZH2/SUZ12) show the most distinct pattern in DNA methylation variation, being furthest away from other chromatin factors in the plot. Providing a more detailed explanation for this divergence would be preferred.
4. Consider how binarization might affect the epiCHAOS scores, as it can exaggerate the impact of varying factor expression levels, especially within specific genomic loci classes. This could lead to overestimations or distortions in the perceived biological heterogeneity.

**Authors Response**

**Point-by-point responses to the reviewers’ comments:**

Reviewer #1:

Kelly et al. proposed a concise metric, epiCHAOS, to evaluate epigenomic heterogeneity in certain singlecell clusters or groups, and applied this metric to multiple single-cell datasets to associate it with cellular phenotypes, such as stemness and plasticity, and genomic features. Although epiCHAOS reflects certain associations with some biological features, there are some limitations which should be addressed when it is widely used in single-cell data routine analyses in future.

*Authors’ response: We thank the reviewer for their constructive comments and we hope that we have adequately addressed the limitations which they have noted below.*

Reviewers comment 1: Although epiCHAOS has been applied in many single-cell datasets, the conclusions are highly dependent on prior knowledge. Are there any examples that we could gain new insights into some biological processes by utilizing epiCHAOS? It is more meaningful that a method could solve some problems or bring some inspirations when used in single-cell routine analyses.

*Authors’ response: We appreciate the reviewer’s comments, and we hope that epiCHAOS should ultimately be used by the scientific community to obtain new biological insights rather than to validate existing paradigms. In this manuscript, we intentionally focused on applications where there was some prior knowledge or expectation in order to demonstrate that the epiCHAOS score is a reliable metric which leads to results that make biological sense. We have clarified this intention in the text (line 132-133). This means that many of our observations - for example, the increased heterogeneity in developmentally plastic tissues, and at polycomb-targeted regions - are backed up by prior knowledge/expectation, which we consider promising. Nevertheless, our findings are novel in the sense that they have never been shown in this kind of data at the single-cell level before.*

*Regarding the future applicability of epiCHAOS, we have suggested several examples where our method might help to yield new biological insights. We have elaborated on this point in the discussion (line 307317) to provide some more concrete recommendations which we hope will inspire future applications. For example, epiCHAOS could be used to investigate the propensity of therapeutic resistance in relationship to epigenetic heterogeneity or whether metastatic cells are epigenetically more heterogeneous than primary tumors. Notably, the datasets required to answer these questions are not yet available, but epiCHAOS could be an essential tool for answering them.*

*Adjustments to the manuscript:*

*To the Results section, we have added “In order to show that epiCHAOS is a reliable metric which leads to biologically plausible results, we initially focused on applications where there was some prior knowledge or expectation.” (line 132-133).*

*To the Discussion we added “EpiCHAOS could be used for example to investigate how epigenetic heterogeneity influences therapeutic resistance, or whether metastatic cells are epigenetically more heterogeneous than primary tumors.” (line 309-311).*

Reviewers comment 2: A epiCHAOS score is calculated for one single-cell cluster or group. It means that this metric is largely dependent on the clustering precision of single cells. However, the single cell clustering is unsupervised and determined by experience. When a large cell cluster is further clustered into some more homologous sub-clusters, will epiCHAOS alter? How to balance epiCHAOS and clustering precision?

*Authors’ response: We thank the reviewer for this valid concern. To address this, we have decided to explore how epiCHAOS behaves under different clustering resolutions, and we have now included this analysis in the manuscript (Figure S3, Figure S4 and Supplementary Note 2). We selected as an example the breast cancer dataset used in Figure 3, where we performed clustering in ArchR using a range of clustering resolutions from 0.1 to 0.9, and compared the resulting epiCHAOS scores. Here we can show that a similar pattern of epiCHAOS scores emerges in different cell groups regardless of clustering resolution (Figure S3), and we detect a high correlation of epiCHAOS scores per-single-cells at different resolutions (Figure S4). This indicates that our metric is minimally dependent on the strategy used for clustering or defining cell groups. As expected, we do find that splitting a large cluster into several smaller clusters can reveal which parts of that larger cluster are more heterogeneous. For example, in this dataset, when the lowest clustering resolution is used, a high epiCHAOS score is assigned to cluster 4. At higher resolutions, this larger cluster is split into five smaller clusters, four of which continue to be assigned high epiCHAOS scores, but one of which is found to be less heterogeneous.*

*As another example of this, we have already explored how epiCHAOS scores vary across different tissue types, and across the various cell types of which those tissues are composed (Figure S5). Here, per-tissue comparison revealed the highest epigenetic heterogeneity in neural tissues and placenta, and when we separated each tissue into its respective cell types, we found that many but not all of the most heterogeneous cell types were also of placental or neural origin.*

*We hope that these additions will clarify the extent to which epiCHAOS can be affected by clustering parameters, and will reassure the reviewer that this effect is minimal. Ultimately, we believe that the choice of how to define clusters or groups of cells should be up to the user and will depend on the questions of the particular study. It is also likely that epiCHAOS will more often be applied to compare known biological conditions/states rather than computationally-defined clusters. Throughout this manuscript we have not made or suggested any changes to the standard, state-of-the-art clustering approaches in this field, and it is beyond the purpose of epiCHAOS to make such recommendations.*

*Adjustments to the manuscript: We added Supplementary Figure S3 to illustrate how epiCHAOS scores change across clusters when clustering resolution is adjusted. We added Supplementary Figure S4 to show the per-single-cell correlation of epiCHAOS scores assigned using different clustering resolutions.*

*To the Results, we added “...and is minimally influenced by differences in clustering resolution (Additional file 1: Figure S3, Figure S4, Additional file 2: Supplementary Note 2).” (line 128-130).*

*We added the following Supplementary Note 2, to comment on this result: “To investigate how epiCHAOS behaves under different clustering parameters, we selected as an example the breast cancer dataset from Kumegawa et al. which we used in Figure 3. We performed clustering in ArchR using a range of clustering resolutions from 0.1 to 0.9. We then computed epiCHAOS scores at each different clustering resolution and compared the results. While we do find that splitting a large cluster into several smaller clusters can reveal which parts of that larger cluster are more heterogeneous than others, the overall results change minimally. We find that a similar pattern of epiCHAOS scores emerges in different cell groups regardless of clustering resolution (Figure S3), with a high correlation of per-single cell epiCHAOS scores measured at different resolutions (Figure S4). This suggests that epiCHAOS scores are minimally influenced by the choice of clustering parameters.”*

Reviewers comment 3: In this manuscript, the authors tended to compare epiCHAOS scores between different cell clusters. In another dimension, they could compare epiCHAOS scores between different samples within the same single-cell clusters or cell types. For example, analyzing datasets associated with aging or drug sensitivity or disease severity.

*Authors’ response: We thank the reviewer for this valuable suggestion. It is true that in the manuscript we mostly performed comparisons between cell types or clusters, however, we did not exclusively focus on such comparisons. Note that we have also used epiCHAOS to compare epigenetic heterogeneity in breast cancer cells sensitive/resistant to capecitabine, where we find increased heterogeneity in therapy-resistant cells (Figure 5D, line 256-259).*

*As suggested, we have also included one further example in the context of aging (Figure 3E). For this, we obtained scATAC-seq data from a previous study of aged and young mouse hematopoietic stem cells, which we used to show that epigenetic heterogeneity is increased in aged cells compared to their younger counterparts. We hope that these additional analyses will help to showcase the utility of epiCHAOS beyond cell type/cluster-level comparisons.*

*Adjustments to the manuscript:*

*We added Figure 3E; a comparison of epiCHAOS in young and aged hematopoietic stem cells.*

*We added “... and aging” to the title of section 3 (line 166).*

*We added the following paragraph to section 3: “We also wondered whether epiCHAOS would detect increased epigenetic heterogeneity in aged tissues. Using a scATAC-seq dataset from young (2 months) and old (24 months) mouse HSCs^38^, epiCHAOS detected a global increase in epigenetic heterogeneity in the aged cells (Figure 3E, Additional file 3: Table S10), reflecting the stochastic loss of epigenetic information which is considered a hallmark of aging^39^.” (line 194-198).*

*We added “... as well as aging in the hematopoietic system.” (line 201).*

*To the discussion, we added “We also find that epigenetic heterogeneity is increased with age in the hematopoietic system, reflecting the expected stochastic decay of epigenetic regulation” (284-285). The resulting scores are added to Supplementary Table S10.*

Reviewers comment 4: P values should be provided for regression or correlation in Fig. 2D-F, Fig. 3 BC, Fig. 4A, D, Fig. 5B.

*Authors’ response: Thank you for pointing this out, we have now added p-values for all correlation analyses. Note that Fig. 4A and 4D depict ranked epiCHAOS scores for different genomic regions (A) and gene sets (D) rather than correlations, so they do not have associated p-values.*

*Adjustments to the manuscript: We added p-values directly to Fig. 2D-F, Fig. 5B, and to the supplementary tables relating to Fig. 3B-C.*

Reviewers comment 5: Another possible utilization for epiCHAOS: could epiCHAOS score be used for guiding single cell clustering and evaluating the similarity across different clusters?

*Authors’ response: We thank the reviewer for pointing out this additional functionality for epiCHAOS. We are aware that previously established methods designed for evaluating single-cell clustering, such as the silhouette score, rely on similar principles as epiCHAOS. Within this manuscript, we specifically focus on the assessment of epigenomic heterogeneity, which requires standard processing of epigenomic data using established tools such as Seurat or ArchR. While we do believe that epiCHAOS might be useful for determining clustering resolution (see also Response to comment #2), we consider it beyond the scope of the current manuscript. We have mentioned this in our discussion as a potential future application.*

*Adjustments to the manuscript:*

*To the discussion, we added: “Moreover, epiCHAOS might also inspire strategies for guiding single-cell clustering and evaluating the similarity/variability across clusters in single-cell epigenomics data.” (line 311-313).*

Reviewers comment 6: It is difficult to determine the difference significance between two clusters by solely comparing two epiCHAOS scores. The authors could introduce P values when comparing two clusters in the method. A possible solution is single cell shuffling across clusters.

*Authors’ response: We thank the reviewer for this important comment to improve the significance of our findings. Recognizing this limitation, we provide two possible solutions:*

*One option that would allow us to assess statistical significance is to use permutation tests (we understand this is what is meant by “single cell shuffling across clusters”). Note that we have already used this approach for differential heterogeneity analysis across different genomic regions (Figure 4F, Figure S9C-D), and we agree with the reviewer that a similar approach may be used for simple genome-wide comparison between* *any two groups. Another option, instead of calculating a single epiCHAOS score for each group/cluster of cells, is to take several subsamples of cells from each group, which will allow for a more robust comparison between groups.*

*We note that the permutation approach is considerably more time consuming given the large amount of permutations required to assess statistical significance. While we included this as an option in the epiCHAOS R package, we decided to use the subsampling approach for the analyses in the manuscript (Figures 2, 3, 5). We have repeated analyses in the manuscript with this pseudo-replicate approach, taking five subsamples of 100 cells from each group, rather than assigning a single epiCHAOS score per group.*

*Adjustments to the manuscript:*

*We have repeated epiCHAOS analyses using the above subsampling approach and have accordingly replaced bar plots in Figures 2A-C and 3D with violin plots. Downstream correlation analyses which used epiCHAOS scores (Figure 2D-F, Figure 3B-C) were also repeated, replacing the epiCHAOS score per group/cluster with the mean of subsample epiCHAOS scores per group/cluster. EpiCHAOS scores reported in supplementary tables were replaced accordingly.*

*We have adjusted the Methods section accordingly: “To allow a more robust comparison between groups, epiCHAOS scores were calculated on five random subsamples of 100 cells from each group/cluster, except in groups/clusters which contained fewer than 100 cells. Since the scNMT-seq data contained fewer than 100 cells in most groups, epiCHAOS scores were calculated only once for each cell type.” (line 354-358).*

Reviewer #2:

The paper provides a novel metric to measure epigenetic heterogeneity, the epiCHAOS score. This score is calculated as the mean pairwise distance (e.g. the Jaccard distance for binary scATAC-seq) between cells within a defined population (e.g., a cell cluster), representing the average dissimilarity or heterogeneity among the cells in that cluster. This score is then adjusted for technical factors, such as differences in genome-wide chromatin accessibility, to ensure that the heterogeneity reflects biological variation regardless of sequencing depth. The validation of epiCHAOS across various contexts—including less differentiated, multipotent cell states in human hematopoiesis, mouse gastrulation, and Drosophila embryogenesis; breast and liver cancer patients; and specific gene sets—demonstrates a comprehensive and well-rounded biological argument of linking epigenetic heterogeneity to key biological processes across different contexts. The ability of epiCHAOS to reveal elevated heterogeneity at PRC2 targets, CTCF, and Cohesin binding sites further strengthens its application in understanding epigenetic regulation. Some comments are still needed, and are listed below:

*Authors’ response: We appreciate the reviewer’s overall positive evaluation of the manuscript. We especially agree with the reviewer that epiCHAOS can be used across various biological datasets and research questions. The remaining concerns will be addressed in the following with a point-by-point answer.*

Reviewers comment 1: The current approach of generating heterogeneous datasets (Fig 1B) (by incrementally removing a set number of "1's" from selected rows and adding them to different rows) introduces a circular problem. Since the Jaccard distance compares the number of shared elements to the total number of unique elements across the sets, manipulating the matrix in this way could artificially influence the metric, potentially leading to results that reflect the method's design rather than real-world biological variations. It would be more effective to focus on in silico simulations that incorporate more biologically relevant models, or by adapting tools for simulation that better mimic the diversity and complexity of real-world data.

*Authors’ response and modification to the manuscript: We appreciate this comment and we agree that this approach does not reflect real-world biological heterogeneity. Nevertheless, we have found this result useful as a way to demonstrate that the chosen metric behaves as expected in completely synthetic data where cell-to-cell differences are incrementally increased. Still, we do not want to overstate the biological relevance of this result, and we want to point out that this is only one of several strategies which we have used to successfully validate our method. Following this initial validation, we applied epiCHAOS to semisynthetic mixtures of cell types, which also yielded the expected results (Figure 1E). Further validation came in applying epiCHAOS to real biological contexts in which we have prior biological expectations, for example in developmental systems (Figure 2) and in hematopoietic aging (newly added, Figure 3E), and in comparison across genomic regions (Figure 4). We believe that the validity of our metric for quantifying real-world biological variations is most strongly supported by the fact that our findings align with existing biological knowledge in all of the above scenarios.*

*As suggested, we have now also made use of more sophisticated in silico simulation strategies that better mimic real-world data, to assess the effect of sequencing depth (see comment 2 below).*

*We modified our description of this result to clarify our intention and to highlight that the data we created are not intended to reflect biologically relevant heterogeneity: “To validate the design of epiCHAOS we generated a range of fully synthetic scATAC-seq datasets in which we controlled the levels of heterogeneity, while maintaining a stable total count (see “Methods” for details). We showed that the epiCHAOS score is highly correlated with the true controlled, artificial heterogeneity.” (line 94-96)*

Reviewers comment 2: To simulate biological samples with varying sequencing depth (Fig. 1C), it could be valuable to adopt more sophisticated scATAC simulation strategies / tools, such as scReadSim. This will generate synthetic data that more closely mimics real sequencing conditions, incorporating realistic variations in read counts, sequencing errors, and chromatin accessibility distribution patterns. For instance, varying sequencing depth in simulations can better reflect how differences in coverage impact peak detection and data quality.

1. Yan, G., Song, D. & Li, J.J. scReadSim: a single-cell RNA-seq and ATAC-seq read simulator. *Nat Commun* **14**, 7482 (2023). https://doi.org/10.1038/s41467-023-43162-w

*Authors’ response: We thank the reviewer for the valuable recommendation as we were not previously aware of the already established in silico simulation tools. We have now incorporated these methods into our workflow to test the effect of sequencing depth on the epiCHAOS score. We used scReadSim to simulate a range of scATAC-seq matrices with varying sequencing depth, using a dataset from hematopoietic stem cells as baseline. Before sparsity adjustment, as we previously noted in the case of copy number alterations (Figure S1), the raw epiCHAOS score did correlate with sequencing depth, being higher in data with fewer reads. We show that the epiCHAOS score is no longer correlated with sequencing depth after we adjust for data sparsity (Figure 1D).*

*Note that we also adjusted the wording in Figure 1A. where we adjust for sparsity (i.e. the proportion of non-zero counts), rather than adjusting for sequencing depth per se. Figure S2A demonstrates that our score does not correlate with sparsity in simple simulations, though we agree that it is not biologically relevant data. We believe that the additional simulation experiment (Figure 1D, Figure S2B), together with previous investigations in the context of copy number alterations (Figure S1) confirm that the metric is robust to different sources of sparsity that can arise in biological data.*

*Adjustments to the manuscript:*

*We have replaced Figure 1D with a comparison of epiCHAOS scores in simulated datasets of varying sequencing depth. We have added Supplementary Figure S2B to show how epiCHAOS behaved in these* *datasets prior to adjustment for sparsity. We adjusted the wording in Figure 1A, replacing “adjust for sequencing depth” with “adjust for sparsity”.*

*We added “...or due to differences in sequencing depth.” (line 111-112).*

*We added “To assess the effect of sequencing depth, we used the published tool, scReadSim^27^. We found that epiCHAOS was not affected by differences in sequencing depth (Figure 1D).” (line 116-118).*

*We have added the following paragraph to the Methods section to describe this additional analysis: “The scReadSim package was used to simulate scATAC-seq data of varying sequencing depths ^54^. A subset of scATAC-seq data from HSCs from the Granja et al. dataset was used as input^27^. Data were reduced to 10,000 randomly selected peaks for ease of processing. Simulated scATAC-seq matrices comprising each 500 cells were generated with sequencing depth ranging from 50,000 to 100,000 counts, in increments of 10,000. EpiCHAOS scores were calculated across matrices on five subsamples of 100 cells from each condition.” (line 384-389).*

Reviewers comment 3: In Fig 4B, the PRC2 targets (EZH2/SUZ12) show the most distinct pattern in DNA methylation variation, being furthest away from other chromatin factors in the plot. Providing a more detailed explanation for this divergence would be preferred.

*Authors’ response: We agree that the divergence of PRC2 targets from other chromatin factors is the most striking observation from this experiment, and this deserves to be commented on in more detail. We have already highlighted in the text that PRC2 targets exhibit the highest heterogeneity both in DNA methylation and chromatin accessibility (line 209-210). We have now expanded on this point in our discussion, where we mention a number of previous studies (Kar et al., Kumar et al., Faure et al.) which similarly reported increased heterogeneity in these regions. We speculate that this may be related to the fact that many PRC2 targets lie within bivalent chromatin, which exhibits both activating and repressive histone marks, and which has been shown to display increased transcriptional noise. As we note in our discussion, others have shown that PRC2 targets exhibit a low transcriptional burst frequency compared to other genes, and that this gives rise to oscillations between on and off states over time. We think this is a reasonable explanation for the especially high epigenetic heterogeneity detected by epiCHAOS in these regions.*

*Adjustments to the manuscript:*

*We extended paragraph 2 of the Discussion to comment on this in more detail: “Our findings regarding the preferential heterogeneity of specific genomic regions – in particular, the elevated cell-to-cell heterogeneity of polycomb binding sites – is also in agreement with previous studies of transcriptional noise and DNA methylation-based heterogeneity. For example, Kar et al. have previously shown that PRC2 targets display higher cell-to-cell variation in gene expression, with a low transcriptional burst frequency giving rise to oscillations between on and off states over time ^40^. Kumar et al.^8^ also demonstrated that polycomb target genes are heterogeneously expressed within colonies of pluripotent stem cells. This heterogeneity may be due to the association of PRC2 targets with bivalent chromatin states – characterized by the presence of both active and repressive histone marks. Faure et al. ^45^ also previously showed that genes with bivalent chromatin exhibit higher transcriptional noise. Similarly, Feinberg & Irizarry have demonstrated that DNA methylation stochasticity is increased at developmental genes – many of which are also PRC2 targets ^46^.” (line 289-299).*

Reviewers comment 4: Consider how binarization might affect the epiCHAOS scores, as it can exaggerate the impact of varying factor expression levels, especially within specific genomic loci classes. This could lead to overestimations or distortions in the perceived biological heterogeneity.

*Authors’ response: We thank the reviewer for their valid concern. While we recognize that this may be a limitation of epiCHAOS in certain scenarios, we note that the sparsity of single-cell epigenomics data would make it particularly challenging to quantify heterogeneity from the data in a continuous form. Binarization of scATAC-seq data is generally expected to reduce overall noise and minimize the effect of data sparsity. We therefore specifically designed our metric for application to binary data, since it is standard practice to handle single-cell epigenomics data in this form. We have added a comment on this in our discussion.*

*Adjustments to the manuscript:*

*To the discussion we added “The sparsity of single-cell epigenomics data makes it particularly challenging to analyze and interpret in a continuous form, and binarization has become standard practice as a way to minimize the effect of sparsity and to reduce overall noise. We therefore specifically designed epiCHAOS for application to binary data. A limitation of this approach is that information about quantitative differences in accessibility between cells is lost, such that certain patterns of biological heterogeneity might be obscured. Nevertheless…” (line 274-278).*

**Second round of review**

**Reviewer 1**

I would like to thank the authors for the revision and my concerns have been addressed.

**Reviewer 2**

We appreciate the authors' detailed response and efforts to improve their manuscript and method. They have made significant improvements in addressing the concerns raised in the initial review by refining their validation approach and clarifying key findings.

In the previous review, we pointed out the limitation of the initial simulation strategy, noting that it introduces a circular problem by design, which artificially influences the Jaccard distance. The authors have addressed this by incorporating in silico simulations using scReadSim, which relies on real datasets as inputs (e.g., BAM files, reference genomes), allowing synthetic reads to better mimic real biological data. For instance, in Figure 1D, they applied scReadSim using a subset of scATAC-seq data from hematopoietic stem cells (HSCs) to simulate varying sequencing depths. They demonstrated that the sparsity adjustment step ensures that the epiCHAOS metric remains robust to variations in sequencing depth.

Regarding point 3 from previous review, the authors provided a more comprehensive explanation of the elevated heterogeneity observed in Figure 4B. They offered a detailed discussion of the increased variability at PRC2 target regions, supported by several lines of experimental evidence and hypotheses. Specifically, they attributed this variability to bivalent chromatin states and increased transcriptional noise, particularly in pluripotent stem cells and developmental genes, referencing a list of studies that reinforce these observations. This expansion strengthens their argument and adds valuable context to their findings.

Overall, with these revisions, we believe the manuscript has been significantly strengthened and will be updating our score accordingly. We commend the authors on their thorough responses and adjustments.
